# (*E*)-1-(1,3-Benzodioxol-5-yl)-3-(3-bromo­phen­yl)prop-2-en-1-one

**DOI:** 10.1107/S1600536808037446

**Published:** 2008-11-20

**Authors:** Hongqi Li, T. V. Sreevidya, B. Narayana, B. K. Sarojini, H. S. Yathirajan

**Affiliations:** a Key Laboratory of Science and Technology of Eco-Textiles, Ministry of Education, College of Chemistry, Chemical Engineering and Biotechnology, Donghua University, Shanghai 201620, People’s Republic of China; b Department of Studies in Chemistry, Mangalore University, Mangalagangotri 574 199, India; c Department of Chemistry, P. A. College of Engineering, Mangalore 574 153, India; d Department of Studies in Chemistry, University of Mysore, Manasagangotri, Mysore 570 006, India

## Abstract

In the title compound, C_16_H_11_BrO_3_, the mol­ecules adopt an *E* configuration with respect to the C=C double bond of the propenone unit. The 13 non-H atoms of the benzodioxole and propenone units are approximately coplanar (r.m.s. deviation = 0.027 Å) and the bromo­benzene ring plane forms a dihedral angle of 10.8 (1)° to this plane. The structure is layered, with the mol­ecules forming a herring-bone arrangement within each layer.

## Related literature

For the use of chalcones as starting materials in the preparation of various mol­ecules including fused heterocyclic compounds, see: Insuasty *et al.* (1997 ▶). For related structures, see: Butcher *et al.* (2007*a* ▶,*b* ▶,*c* ▶); Low *et al.* (2002 ▶).
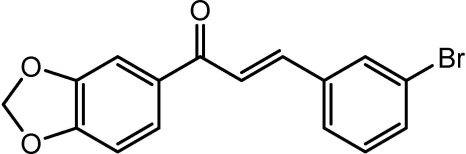


## Experimental

### 

#### Crystal data

C_16_H_11_BrO_3_
*M* *_r_* = 331.16
Monoclinic, 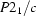

*a* = 14.237 (3) Å
*b* = 8.1811 (17) Å
*c* = 11.717 (2) Å
β = 100.658 (3)°
*V* = 1341.1 (5) Å^3^
*Z* = 4
Mo *K*α radiation
μ = 3.07 mm^−1^
*T* = 273 (2) K
0.12 × 0.10 × 0.06 mm

#### Data collection

Bruker SMART APEXII CCD diffractometer
Absorption correction: multi-scan (*SADABS*; Bruker, 2005 ▶) *T*_min_ = 0.710, *T*_max_ = 0.837
6752 measured reflections
2362 independent reflections
1869 reflections with *I* > 2σ(*I*)
*R*_int_ = 0.021

#### Refinement

*R*[*F* ^2^ > 2σ(*F* ^2^)] = 0.026
*wR*(*F* ^2^) = 0.073
*S* = 1.04
2362 reflections
182 parameters
H-atom parameters constrained
Δρ_max_ = 0.35 e Å^−3^
Δρ_min_ = −0.29 e Å^−3^

### 

Data collection: *APEX2* (Bruker, 2005 ▶); cell refinement: *SAINT* (Bruker, 2005 ▶); data reduction: *SAINT*; program(s) used to solve structure: *SHELXS97* (Sheldrick, 2008 ▶); program(s) used to refine structure: *SHELXL97* (Sheldrick, 2008 ▶); molecular graphics: *SHELXTL* (Sheldrick, 2008 ▶); software used to prepare material for publication: *SHELXTL*.

## Supplementary Material

Crystal structure: contains datablocks global, I. DOI: 10.1107/S1600536808037446/bi2315sup1.cif

Structure factors: contains datablocks I. DOI: 10.1107/S1600536808037446/bi2315Isup2.hkl

Additional supplementary materials:  crystallographic information; 3D view; checkCIF report

## Figures and Tables

**Fig. 1.**
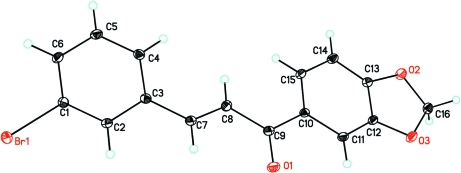
Molecular structure of the title compound with displacement ellipsoids drawn at the 30% probability level for non-H atoms.

## supplementary crystallographic information

### Comment

Chalcones have been widely used as starting materials in preparation of various molecules including fused heterocyclic compounds (Insuasty *et al.*, 1997). Chalcones are also finding application as organic nonlinear optical (NLO) materials because of their SHG conversion efficiency. The crystal structures of some benzodioxol- and bromo-substituted chalcones have been studied (Butcher *et al.*, 2007*a*,*b*,*c*). In continuation of this theme, and also owing to the general importance of these flavanoid analogues, we report herein the synthesis and crystal structure of a new chalcone, namely (2*E*)-1-(1,3-benzodioxol-5-yl)- 3-(3-bromophenyl)prop-2-en-1-one.

### Experimental

To a mixture of 1-(1,3-benzodioxol-5-yl)ethanone (1.64 g, 0.01 mol) and 3-bromobenzaldehyde (1.86 g, 0.01 mol) in 25 ml of ethanol, 50% KOH(aq) was added. The mixture was stirred for one hour at room temperature then the precipitate was collected by filtration and purified by recrystallization from ethanol. Single crystals were grown from toluene by slow evaporation. Yield: 82 %, m.p. 393–395 K. Elemental analysis calculated: C 58.03, H 3.35%; found: C 58.12, H 3.21%.

### Refinement

H atoms were placed at calculated positions and refined using a riding model, with C—H = 0.93–0.97 Å and *U*_iso_(H) = 1.2*U*_eq_(C).

### Crystal data

**Table:**

C_16_H_11_BrO_3_	*F*_000_ = 664
*M**_r_* = 331.16	*D*_x_ = 1.640 Mg m^−^^3^
Monoclinic, *P*2_1_/*c*	Mo *K*α radiation λ = 0.71073 Å
Hall symbol: -P 2ybc	Cell parameters from 3007 reflections
*a* = 14.237 (3) Å	θ = 2.9–28.0º
*b* = 8.1811 (17) Å	µ = 3.07 mm^−^^1^
*c* = 11.717 (2) Å	*T* = 273 (2) K
β = 100.658 (3)º	Block, colorless
*V* = 1341.1 (5) Å^3^	0.12 × 0.10 × 0.06 mm
*Z* = 4	

### Data collection

**Table:**

Bruker SMART APEXII CCD diffractometer	2362 independent reflections
Radiation source: fine-focus sealed tube	1869 reflections with *I* > 2σ(*I*)
Monochromator: graphite	*R*_int_ = 0.021
*T* = 273(2) K	θ_max_ = 25.0º
φ and ω scans	θ_min_ = 2.9º
Absorption correction: multi-scan(SADABS; Bruker, 2001)	*h* = −16→16
*T*_min_ = 0.710, *T*_max_ = 0.837	*k* = −9→9
6752 measured reflections	*l* = −13→9

### Refinement

**Table:**

Refinement on *F*^2^	Hydrogen site location: inferred from neighbouring sites
Least-squares matrix: full	H-atom parameters constrained
*R*[*F*^2^ > 2σ(*F*^2^)] = 0.026	*w* = 1/[σ^2^(*F*_o_^2^) + (0.0383*P*)^2^ + 0.3973*P*] where *P* = (*F*_o_^2^ + 2*F*_c_^2^)/3
*wR*(*F*^2^) = 0.073	(Δ/σ)_max_ = 0.001
*S* = 1.04	Δρ_max_ = 0.35 e Å^−^^3^
2362 reflections	Δρ_min_ = −0.29 e Å^−^^3^
182 parameters	Extinction correction: SHELXL97 (Sheldrick, 2008)
Primary atom site location: structure-invariant direct methods	Extinction coefficient: 0.0063 (7)
Secondary atom site location: difference Fourier map	

### Special details

**Table:**

Geometry. All e.s.d.'s (except the e.s.d. in the dihedral angle between two l.s. planes) are estimated using the full covariance matrix. The cell e.s.d.'s are taken into account individually in the estimation of e.s.d.'s in distances, angles and torsion angles; correlations between e.s.d.'s in cell parameters are only used when they are defined by crystal symmetry. An approximate (isotropic) treatment of cell e.s.d.'s is used for estimating e.s.d.'s involving l.s. planes.
Refinement. Refinement of *F*^2^ against ALL reflections. The weighted *R*-factor *wR* and goodness of fit *S* are based on *F*^2^, conventional *R*-factors *R* are based on *F*, with *F* set to zero for negative *F*^2^. The threshold expression of *F*^2^ > σ(*F*^2^) is used only for calculating *R*-factors(gt) *etc*. and is not relevant to the choice of reflections for refinement. *R*-factors based on *F*^2^ are statistically about twice as large as those based on *F*, and *R*-factors based on ALL data will be even larger.

### Fractional atomic coordinates and isotropic or equivalent isotropic displacement parameters (Å^2^)

**Table:**

	*x*	*y*	*z*	*U*_iso_*/*U*_eq_	
Br1	0.489230 (17)	1.25202 (3)	0.29468 (2)	0.05206 (13)	
O1	0.93644 (14)	0.7277 (2)	0.27018 (16)	0.0622 (6)	
O2	1.29937 (12)	0.4896 (2)	0.61302 (13)	0.0541 (5)	
O3	1.26700 (12)	0.4663 (3)	0.41361 (14)	0.0599 (5)	
C1	0.58905 (15)	1.1426 (3)	0.39752 (18)	0.0360 (5)	
C2	0.65910 (15)	1.0623 (3)	0.35360 (18)	0.0368 (5)	
H2	0.6575	1.0608	0.2739	0.044*	
C3	0.73304 (16)	0.9827 (3)	0.42861 (18)	0.0370 (5)	
C4	0.73303 (17)	0.9887 (3)	0.54787 (19)	0.0433 (6)	
H4	0.7819	0.9381	0.5994	0.052*	
C5	0.66100 (17)	1.0691 (3)	0.58983 (19)	0.0455 (6)	
H5	0.6615	1.0709	0.6693	0.055*	
C6	0.58831 (16)	1.1468 (3)	0.51479 (19)	0.0427 (6)	
H6	0.5399	1.2009	0.5430	0.051*	
C7	0.80648 (16)	0.8953 (3)	0.3795 (2)	0.0424 (6)	
H7	0.7946	0.8819	0.2993	0.051*	
C8	0.88707 (17)	0.8340 (3)	0.4361 (2)	0.0464 (6)	
H8	0.9018	0.8455	0.5164	0.056*	
C9	0.95504 (19)	0.7472 (3)	0.3760 (2)	0.0431 (6)	
C10	1.04589 (16)	0.6841 (3)	0.44539 (18)	0.0355 (5)	
C11	1.11052 (16)	0.6051 (3)	0.38609 (19)	0.0427 (6)	
H11	1.0972	0.5928	0.3058	0.051*	
C12	1.19286 (16)	0.5475 (3)	0.45046 (18)	0.0386 (5)	
C13	1.21294 (16)	0.5615 (3)	0.56996 (19)	0.0394 (5)	
C14	1.15146 (17)	0.6343 (3)	0.6304 (2)	0.0493 (6)	
H14	1.1650	0.6418	0.7109	0.059*	
C15	1.06741 (18)	0.6969 (3)	0.5658 (2)	0.0418 (6)	
H15	1.0242	0.7489	0.6042	0.050*	
C16	1.33689 (17)	0.4360 (3)	0.5143 (2)	0.0473 (6)	
H16A	1.3952	0.4951	0.5098	0.057*	
H16B	1.3516	0.3203	0.5208	0.057*	

### Atomic displacement parameters (Å^2^)

**Table:**

	*U*^11^	*U*^22^	*U*^33^	*U*^12^	*U*^13^	*U*^23^
Br1	0.03784 (17)	0.0701 (2)	0.04670 (18)	0.00990 (12)	0.00367 (11)	0.00788 (12)
O1	0.0524 (12)	0.0986 (16)	0.0368 (11)	0.0217 (10)	0.0111 (8)	0.0093 (9)
O2	0.0400 (10)	0.0774 (12)	0.0419 (9)	0.0128 (9)	−0.0005 (7)	−0.0046 (9)
O3	0.0465 (10)	0.0928 (14)	0.0404 (9)	0.0245 (10)	0.0086 (8)	−0.0085 (9)
C1	0.0279 (11)	0.0424 (13)	0.0372 (12)	−0.0039 (9)	0.0045 (9)	0.0031 (9)
C2	0.0360 (12)	0.0434 (13)	0.0317 (11)	−0.0044 (10)	0.0080 (9)	0.0011 (9)
C3	0.0351 (12)	0.0400 (12)	0.0361 (12)	−0.0047 (10)	0.0069 (9)	0.0021 (10)
C4	0.0381 (13)	0.0520 (14)	0.0383 (12)	0.0012 (11)	0.0034 (10)	0.0063 (11)
C5	0.0462 (14)	0.0609 (16)	0.0301 (12)	−0.0019 (12)	0.0090 (10)	−0.0019 (11)
C6	0.0352 (13)	0.0548 (15)	0.0403 (13)	0.0008 (11)	0.0128 (10)	−0.0035 (11)
C7	0.0419 (14)	0.0489 (14)	0.0382 (13)	0.0028 (11)	0.0127 (10)	0.0068 (10)
C8	0.0439 (14)	0.0554 (15)	0.0406 (13)	0.0076 (12)	0.0101 (11)	−0.0007 (11)
C9	0.0440 (14)	0.0498 (14)	0.0375 (14)	0.0025 (11)	0.0129 (10)	0.0058 (10)
C10	0.0352 (12)	0.0384 (11)	0.0347 (12)	−0.0018 (10)	0.0113 (9)	0.0034 (10)
C11	0.0437 (14)	0.0549 (15)	0.0300 (12)	0.0041 (11)	0.0081 (10)	0.0013 (10)
C12	0.0352 (12)	0.0470 (13)	0.0356 (12)	0.0019 (10)	0.0115 (10)	−0.0016 (10)
C13	0.0329 (12)	0.0474 (13)	0.0367 (12)	−0.0029 (10)	0.0031 (9)	−0.0013 (10)
C14	0.0473 (15)	0.0695 (17)	0.0300 (12)	0.0052 (13)	0.0042 (11)	−0.0067 (11)
C15	0.0412 (14)	0.0499 (13)	0.0369 (13)	0.0013 (11)	0.0142 (10)	−0.0034 (10)
C16	0.0366 (13)	0.0539 (15)	0.0504 (14)	0.0058 (11)	0.0056 (11)	−0.0028 (11)

### Geometric parameters (Å, °)

**Table:**

Br1—C1	1.908 (2)	C7—C8	1.314 (3)
O1—C9	1.230 (3)	C7—H7	0.930
O2—C13	1.373 (3)	C8—C9	1.479 (3)
O2—C16	1.429 (3)	C8—H8	0.930
O3—C12	1.382 (3)	C9—C10	1.487 (3)
O3—C16	1.418 (3)	C10—C15	1.391 (3)
C1—C2	1.371 (3)	C10—C11	1.408 (3)
C1—C6	1.376 (3)	C11—C12	1.356 (3)
C2—C3	1.401 (3)	C11—H11	0.930
C2—H2	0.930	C12—C13	1.381 (3)
C3—C4	1.398 (3)	C13—C14	1.360 (3)
C3—C7	1.469 (3)	C14—C15	1.390 (3)
C4—C5	1.383 (3)	C14—H14	0.930
C4—H4	0.930	C15—H15	0.930
C5—C6	1.383 (3)	C16—H16A	0.970
C5—H5	0.930	C16—H16B	0.970
C6—H6	0.930		
			
C13—O2—C16	106.13 (17)	O1—C9—C10	120.6 (2)
C12—O3—C16	106.43 (17)	C8—C9—C10	119.1 (2)
C2—C1—C6	121.8 (2)	C15—C10—C11	119.6 (2)
C2—C1—Br1	119.75 (16)	C15—C10—C9	122.3 (2)
C6—C1—Br1	118.45 (17)	C11—C10—C9	118.13 (19)
C1—C2—C3	120.1 (2)	C12—C11—C10	117.5 (2)
C1—C2—H2	119.9	C12—C11—H11	121.3
C3—C2—H2	119.9	C10—C11—H11	121.3
C4—C3—C2	118.1 (2)	C11—C12—C13	122.1 (2)
C4—C3—C7	122.7 (2)	C11—C12—O3	128.7 (2)
C2—C3—C7	119.15 (19)	C13—C12—O3	109.26 (19)
C5—C4—C3	120.6 (2)	C14—C13—O2	128.0 (2)
C5—C4—H4	119.7	C14—C13—C12	122.1 (2)
C3—C4—H4	119.7	O2—C13—C12	109.89 (19)
C6—C5—C4	120.7 (2)	C13—C14—C15	116.7 (2)
C6—C5—H5	119.7	C13—C14—H14	121.6
C4—C5—H5	119.7	C15—C14—H14	121.6
C1—C6—C5	118.7 (2)	C14—C15—C10	122.0 (2)
C1—C6—H6	120.7	C14—C15—H15	119.0
C5—C6—H6	120.7	C10—C15—H15	119.0
C8—C7—C3	127.3 (2)	O3—C16—O2	108.07 (18)
C8—C7—H7	116.3	O3—C16—H16A	110.1
C3—C7—H7	116.3	O2—C16—H16A	110.1
C7—C8—C9	122.0 (2)	O3—C16—H16B	110.1
C7—C8—H8	119.0	O2—C16—H16B	110.1
C9—C8—H8	119.0	H16A—C16—H16B	108.4
O1—C9—C8	120.3 (2)		
			
C6—C1—C2—C3	−0.5 (3)	C15—C10—C11—C12	−1.4 (3)
Br1—C1—C2—C3	179.54 (16)	C9—C10—C11—C12	−179.7 (2)
C1—C2—C3—C4	−0.3 (3)	C10—C11—C12—C13	1.4 (4)
C1—C2—C3—C7	179.0 (2)	C10—C11—C12—O3	179.9 (2)
C2—C3—C4—C5	1.0 (4)	C16—O3—C12—C11	178.7 (2)
C7—C3—C4—C5	−178.4 (2)	C16—O3—C12—C13	−2.6 (3)
C3—C4—C5—C6	−0.8 (4)	C16—O2—C13—C14	−178.3 (3)
C2—C1—C6—C5	0.7 (3)	C16—O2—C13—C12	3.2 (3)
Br1—C1—C6—C5	−179.37 (17)	C11—C12—C13—C14	−0.2 (4)
C4—C5—C6—C1	0.0 (4)	O3—C12—C13—C14	−179.0 (2)
C4—C3—C7—C8	−10.9 (4)	C11—C12—C13—O2	178.4 (2)
C2—C3—C7—C8	169.8 (2)	O3—C12—C13—O2	−0.4 (3)
C3—C7—C8—C9	179.6 (2)	O2—C13—C14—C15	−179.3 (2)
C7—C8—C9—O1	−1.4 (4)	C12—C13—C14—C15	−1.0 (4)
C7—C8—C9—C10	178.7 (2)	C13—C14—C15—C10	1.0 (4)
O1—C9—C10—C15	−176.9 (2)	C11—C10—C15—C14	0.2 (4)
C8—C9—C10—C15	3.1 (3)	C9—C10—C15—C14	178.5 (2)
O1—C9—C10—C11	1.4 (4)	C12—O3—C16—O2	4.5 (3)
C8—C9—C10—C11	−178.6 (2)	C13—O2—C16—O3	−4.7 (3)
